# Heat Treatment, Cultivar and Formulation Modify the Sensory Properties and Consumer Acceptability of Gels Containing Faba Bean (*Vicia faba* L. *minor*) Protein Concentrates

**DOI:** 10.3390/foods11193018

**Published:** 2022-09-28

**Authors:** Adeline Karolkowski, Christophe Martin, Emilie Bouzidi, Jean-François Albouy, Loïc Levavasseur, Loïc Briand, Christian Salles

**Affiliations:** 1 CSGA (Centre des Sciences du Goût et de l’Alimentation), CNRS, INRAE, Institut Agro, Université de Bourgogne-Franche Comté, 21000 Dijon, France; 2 Groupe Soufflet (Ets J. Soufflet), 10400 Nogent-sur-Seine, France

**Keywords:** faba bean, protein concentrate, heat treatment, off-flavours, off-notes, bitterness, sensory profile, consumer

## Abstract

Faba bean (*Vicia faba* L. *minor*) is an emerging plant-based ingredient due to its environmental, nutritional and functional benefits. However, like other pulses, it exhibits many off-flavours that limit its consumption. Little is known about the flavour of faba beans, and previous studies have focused on volatile compounds and the overall flavour. In the present study, xanthan gum gels were formulated with crude or heated protein concentrates from three faba bean cultivars and with the addition of oil and/or salt. A trained panel analysed the sensory properties of these gels, and a consumer test was carried out to assess their acceptability. The gels formulated with crude protein concentrates exhibited bitterness and green, metallic and rancid notes that decreased consumer appreciation. The heat treatment promoted pleasant notes such as potato, cereal and haricot bean notes and attenuated the most penalising descriptors associated with off-flavours. Cultivar 2 was characterised by fewer off-flavours and should be preferred over the other cultivars for the formulation of faba bean products. This work provides information on the sensory properties of different cultivars of faba bean concentrates and information likely to help improve their acceptability in the context of the formulation of food intended for humans.

## 1. Introduction

Since the 2000s, the world production of dry faba beans has constantly been increasing [1]. Although they are the oldest legumes, faba beans are less cultivated than peas or chickpeas [1,2]. However, faba beans exhibit environmental, nutritional and food functional benefits. Faba bean crops are easier to implement than pea crops and are resistant to certain pests [3,4]. The fixation of atmospheric nitrogen via symbiotic bacteria (rhizobia) into the soil and grain is more important in faba beans than in chickpeas or common beans. The amount of nitrogen in the shoots of the following wheat is higher in faba bean fields than in chickpea, common bean, lupin and pea fields [5]. These benefits reduce the use of nitrogen fertilisers and greenhouse gas emissions [6]. Moreover, faba beans contain more proteins and less starch and lipids than peas, which allow for easy recovery of their proteins [7]. This pulse also exhibits interesting functionalities; it presents foaming, emulsion, water/oil holding and gelling properties and high protein solubility [4,8]. Finally, whole faba beans or derived products such as flour, starch-rich fraction, protein concentrate (dry extraction) and protein isolate (wet extraction) are used for the formulation of novel products or the substitution of ingredients (rich in starch and/or protein) in baked, meat, dairy or pasta products [4,9,10].

In France, faba beans are mainly exploited for animal feed (poultry, pigs and fish) and limited for human consumption due to supply difficulties (lack of visibility on production volumes), price and quality [11]. Moreover, the French population is culturally more accustomed to consuming lentils and chickpeas than faba beans [12]. Like most legumes, they present off-flavours that decrease consumer acceptability [13]. The presence of unpleasant volatile and non-volatile compounds induces the perception of off-flavours in pulses. The flavour is the sum of the aromas, taste and trigeminal sensations. Volatile compounds are responsible for the off-notes (unpleasant aromas) and are described as beany, fatty, green, rancid, earthy, cardboard, pungent, medicinal, beany, pea and brothy [13]. They mainly come from free fatty acid enzymatic oxidation by lipoxygenase (LOX) or autoxidation and free amino acid degradation [13,14]. The occurrence of these reactions is related to cultivar types and the conditions during grain growth, storage and transformation processes [14,15]. A study showed that faba beans have fewer volatile compounds than other pulses, including soybeans, peas, chickpeas, lentils, mung beans, cowpeas, adzuki beans and common beans [16]. Although faba beans are lower in lipids than chickpeas, they exhibit a higher LOX activity that promotes volatile compounds [16,17]. However, the non-volatile compounds naturally present in pulses, such as phenolic compounds, saponins, alkaloids, peptides and free amino acids, could be responsible for the astringency, metallic perception and bitter taste [13,18,19,20,21,22]. Astringent molecules cause the drying, roughening and puckering of the mouth epithelia [23]. The mechanism of astringency is still not well understood. The astringency could be detected by mechanoreceptors in the oral mucosa after the increase of friction forces at the surface of epithelial cells [24,25] or by detection of the aggregation of the mucosa pellicle by the transmembrane mucin (MUC1) [26]. Faba beans are less astringent than red beans, azuki beans, peas and lentils [27]. Metallic perception could be due to salivary protein oxidation by minerals and the production of oxidation-related aldehydes related to odourant lipids [28]. Some mineral ions, such as iron and copper, have been identified in the faba bean [29] and could be indirectly involved in this metallic perception [30]. Bitter taste is induced by compounds that activate bitter taste receptors (TAS2Rs—Taste Receptor 2) located on the tongue and in the oral cavity [23].

Little is known about faba bean sensory properties and thus about its off-flavours. Researchers have focused on the odour profile [31,32] and overall flavour rather than on the identification of the sensory descriptors. The odour profile of aqueous suspensions made with hulled crude and heated faba bean flours was determined. The odours were described as fresh pea, musty, bitter (evoking bitterness), toasty, grassy and peanut, but they were not referred to as food standards. The bitter aroma was more intense in the suspension with crude flour than in those with heated flour [31]. Schultz et al. identified a dried pea-like flavour at neutral pH and a fruity off-flavour at acidic pH in faba bean protein isolate [33]. Eleven whole faba bean lots were described with a beany smell, strong smell, beany “taste”, cooked “taste”, unripe “taste”, bitter aftertaste and lingering “taste” descriptors. The unripe “taste”, bitterness, beany smell and strong smell differed according to the cultivar [34]. Hinchcliffe carried out a sensory profile of faba bean flour and wheat flour slurries. Dried pea and bitterness were the dominant flavours of raw faba bean flour. Moreover, the faba bean concentrate exhibited a higher dried pea flavour and bitterness than the flour and the starch fractions from air classification [35]. However, several sensory analyses of products enriched with faba beans were conducted but were more focused on the colour, texture, overall flavour/aroma/taste and acceptability. For example, the sensory properties of apple juice fortified with faba bean protein hydrolysates, a substitute of semolina with fermented faba bean flour in pasta, meat analogues made with faba bean concentrate and maise bread formulated with faba bean flour were studied [36,37,38,39].

Many strategies have been studied to limit the off-flavours in pulses, such as grain germination, fermentation, defatting, washing, use of flavour masking agents or LOX deactivation by heat treatments, including blanching, roasting, micro-wave, spray-drying, extrusion-cooking or steam heating [30,38,39,40,41,42,43,44,45,46]. Concerning faba bean, defatting, isoelectric washing, or the addition of strawberry aroma reduced the fruity off-flavour of faba bean protein isolate [33]. Heat treatment (70 to 160 °C for 15 to 60 min) significantly decreased the dried pea flavour in faba bean concentrate and flour [35]. Thus, heat treatment should be an interesting way to improve the flavour of faba beans and other pulses.

The present study aimed to characterise the sensory profile of faba beans, identify the perceptions involved in the off-flavours and compare the flavours of three different faba bean protein concentrate cultivars in a food model under their crude (P) and heated (H) forms. For this characterisation, xanthan gum was used for the formulation of a gel food model to get closer to the various food applications made from faba beans (pasta, bread, meat analogues, mayonnaise, cracker and cake). Xanthan gum has many benefits, including its flavourlessness, the formation of gels at ambient temperature and its stability to salts and freezing [40,41]. Moreover, more complex gels were formulated with the addition of salt and/or oil to better simulate plant-based products (for example, pasta or meat analogues). The identification of cultivars that are low in off-flavours or strategies that limit the perception of unpleasant flavours could help increase overall consumer acceptance of faba beans and enable the development of novel food applications.

First, xanthan gum gels made with the addition of heated or crude faba bean protein concentrates were formulated. Then, a trained panel analysed the sensory properties of the gels produced. The sensory profiles of the gels were established based on the intensity of 17 sensory descriptors (4 textures, 3 tastes and 10 aromas). Gel acceptability was also measured using a consumer panel and correlated with the sensory profile data to identify the descriptors responsible for the off-flavours in the faba bean concentrates.

## 2. Materials and Methods

### 2.1. Faba Bean Protein Concentrates

Three cultivars of mature faba bean seeds were provided by Soufflet Agriculture (Groupe Soufflet, Nogent-sur-Seine, France). For each cultivar, protein concentrate was produced by an external laboratory (SAS IMPROVE, Amiens, France) according to the same process used by Groupe Soufflet. They were named P1, P2 and P3. 

A certain quantity of water (Evian®, Evian, France) was added to the protein concentrates. The mix was heat-treated in a drying chamber (Binder^TM^, Tuttlingen, Germany) over a temperature range of 130 to 180 °C and then passed through a sieve with a 400 µm pore size (Retsch, Haan, Germany) to limit aggregate formation. P1, P2 and P3 were heat-treated and named HP1, HP2 and HP3, respectively.

The protein concentrates, P and HP, were vacuum-packed in a glass container and stored at −20 °C before the analyses. The cultivars and heat treatment parameters are not given for confidentiality reasons.

### 2.2. Gel Formulation

The objective was to obtain a gel flavour similar to that of the concentrate alone. A series of internal tastings enabled the formulation of xanthan gels made with protein concentrates which was adapted from [42,43]. A total of 20 g of crude (P) or heated (HP) protein concentrate and 1.0 g of xanthan gum (Texturas, Barcelona, Spain) were mixed (Thermomix TM 5, Vorwerk, Cloyes-sur-le-Loire, France) with 79 g of water (Evian, France) to make a gel at ambient temperature (20 °C). Then, 0.6 g of monosodium glutamate (MSG, 99% pure; Ajinomoto, Tokyo, Japan) was added to the gel to make the flavour more similar to that of the protein concentrate (Figure S1, see Supplementary Materials). For some gels, 0.25% chloride sodium salt (NaCl, Saline Cerebos, Levallois-Perret, France) and/or 3% deodorised sunflower oil (Cœur de Tournesol, Lessieur, Asnière-sur-Seine, France) were added to the previous mix. For all gels, the mixing speed was 500 rpm for 10 min. The different formulations resulted in 12 different samples (Table 1). The samples were stored at −20 °C after formulation and defrosted at 4 °C one day before serving.

### 2.3. Gel Consistency

The consistency of the 12 gels was measured in triplicate at ambient temperature (20 °C). A texture analyser (TA.XT Plus, Stable Micro Systems, Surrey, UK) equipped with a 5-kg load cell was used, following a back-extrusion method [44]. The level of the sample inside the container (height 67 mm, diameter 32 mm) was approximately 50 mm. A 20-mm diameter plastic plate was driven 30 mm into the gel at a constant speed of 0.5 mm/s (pre-test speed 1 mm/s, trigger force 0.01 N). The force of resistance to compression of the gel (N) was determined versus the distance in the sample (mm). The gel consistency (N.mm) was estimated from the average value of the force over the constant part of the curve (10–25 mm) multiplied by the constant part of the curve distance (15 mm). The consistency results are presented in Table 1.

### 2.4. Sensory Analyses

#### 2.4.1. Experimental Conditions

An ethical committee approved this study (Inserm (French National Institute for Health and Medical Research) Ethic Evaluation Committee N° 21-773, approved in February 2021). All panellists signed an informed consent form before the study. Participants with food allergies, especially favism, were excluded from the study.

Both sensory profiling and consumer liking sessions were conducted in an air-conditioned room (20 °C) in separate booths (AFNOR, 1987). Red lights were used to mask potential colour differences. Both panellists and consumers were paid for their participation. The participants answered sensory questionnaires using FIZZ Data Acquisition (Biosystèmes, Couternon, France). For both sensory profiling and consumer sessions, samples were served at 20 °C (room temperature) in transparent cups (30 mL) bearing three-digit codes. After each tasting, participants were requested to take a break of 40 s and to rinse their mouths with water and apples.

#### 2.4.2. Sensory Profiling

The sensory properties were characterised using a conventional sensory profile (AFNOR, 2003). Twenty-one panellists (35–73 years old, 12 females and 9 males) were recruited from Dijon (France) and surrounding areas.

The selection was carried out over two sessions and was based on their availability, their interest in the study, and their sensory abilities, such as taste and olfactory capacities (sensitivity and recognition tests). All panellists already participated in sensory profiling. Training included 14 sessions of a 1-h duration. Panellists were asked not to drink, eat, or use perfume one hour before sensory analysis. The study was carried out over three months. Products presented at each session were chosen to represent the diversity of the product space. Training sessions 1–2 were devoted to descriptor generation. At each session, panellists tasted six of the selected products and generated as many aroma, taste and texture descriptors as they could. The aromas were relative to the retro-nasal perceptions, whereas the tastes included oral perceptions that could be detected with a nose clip. Training sessions 3–9 were focused on identifying the descriptors that discriminated among samples and defined each descriptor with a flavour standard and/or a verbal definition. At the end of this phase, a consensus was reached on 17 descriptors: 4 descriptors of texture, 3 descriptors of taste and 10 descriptors of aroma (Table 2). The descriptors were defined in accordance with the panellists. During sessions 3–12, panellists were also trained in evaluating the intensity of the different descriptors. The intensity was assessed using linear scales, and the score varied from 0 (left of the scale) to 10 (right of the scale). The labels present at the anchors of the scales are specified in Table 2. At each training session, the panellists were asked to evaluate the perceived intensity of the 17 descriptors for 6 randomly selected gels. The training gels were not always similar to those evaluated (Table 1). Sometimes the concentrations of NaCl and MSG were different; the water was partially replaced by pea infusion, and the concentrate used was a mix of the 3 concentrates or originated from an industrial concentrate. Moreover, they could use a nose clip to avoid confusion between salty and umami tastes and broth aroma [45,46]. In this case, they were asked to evaluate descriptor intensity a few seconds after removing the nose clip. At the end of the training, panellist performance was evaluated on eight gels (C1, H1, C2, H2, C3, C3+S+O, H3, H3+S+O) tasted twice during two sessions.

After ensuring the performance of the panel, measurement sessions were carried out. The panellists performed a monadic evaluation of the 12 samples during the same session. The order of the descriptors was the same among the sessions and panellists. The order of presentation of the samples followed a Williams Latin square design [47]. This measurement was repeated once, 2 days after the first.

**Table 2:** Sensory descriptors, evaluated by 21 judges, with their definitions and standards.

Descriptor Groups	Descriptors	Scale Anchors	Definitions	Standards
Texture	Sticky	Not much/very	Sticky feeling perceived between the palate and tongue when manipulating the gel in the mouth.	-
	Thick	Fluid/thick	Thick feeling perceived in the mouth related to the gel viscosity.	-
	Granular	Smooth/granular	Grainy feeling perceived in the mouth related to grains or granules in the gel.	-
	Foamy	Not much/very	Foamy feeling perceived in the mouth related to bubbles in the gel.	-
Tastes *	Salty	Absent/very intense	Basic taste caused by dilute aqueous solutions of various substances such as sodium chloride.	2 g/L of sodium chloride solution (Cooper Standard, Northville, MI, USA).
	Bitter		Basic taste caused by dilute aqueous solutions of various substances such as caffeine.	0.5 g/L of caffeine solution (Cooper Standard, Northville, MI, USA).
	Umami		Basic taste caused by dilute aqueous solutions of various substances such as umami.	0.6 g/L of monosodium glutamate solution (99% pure; Ajinomoto, Tokyo, Japan).
Aromas **	Green note	Absent/very intense	Aromas of peas, French beans, asparagus, green legumes and grass.	Filtered infusion of mashed frozen pea (Carrefour Classic’, Massy, France) in water at 4 °C for 24 h.
	Mushroom		Specific aroma of mushroom.	Filtered infusion of mushroom cooked for 5 min in boiling water.
	Broth		Specific aroma of broth.	Filtered homemade broth with onion, celery, carrot, turnip, leek and clove in water cooked for 1 h.
	Haricot bean		Aromas of haricot bean, dry beans and chickpea.	Juice of haricot bean can (Carrefour^®^, France).
	Cereal		Specific aroma of cereal.	3 g of type 55 wheat flour (Carrefour^®^, France).
	Metallic ***		Specific aroma of metallic.	0.05 g/L of ferrous sulphate (Sigma-Aldrich, St. Quentin Fallavier, France).
	Potato		Specific aroma of potato.	Filtered infusion of potato cooked for 5 min in boiling water.
	Grilled		Specific aroma of grilled.	Filtered infusion of grilled bread (200 °C—10 min) in water.
	Rancid		Specific aroma of rancid.	Piece of a wafer sheet (Modecor Italiana, Cuvio, Italy) exposed to light and dioxygen at ambient temperature.
	Yeast		Aroma of yeast and cheese.	3 g of yeast (Gerblé^®^, France).

* For taste standards, the concentration referred to salty and bitter R1 and half of umami R1 of the SpectrumTM method [48]. ** The aroma descriptors were consistent with those in the pulse sensory wheel made by Chigwedere et al., except for rancid [49]. *** The metallic descriptor was classified in the aroma group, but the metallic perception originated from both volatile and non-volatile compounds (Table S2, Supplementary Materials).

#### 2.4.3. Consumer Liking

Eighty-five consumers with no previous experience in sensory analysis and no self-reported problems with their sense of smell were selected for the study. They were mainly consumers of pulses and pulse-based products more than one time per week, as indicated on a questionnaire on pulse consumption habits. The sociodemographic characteristics and pulse consumption frequency are presented in Table 3. During a 1-h session, the panellists evaluated the 12 samples whose order of presentation followed a Williams Latin square design (Table 1). For each product, consumers had to successively taste the 12 products and give a liking score on a linear scale labelled at each anchor (left anchor: “dislike very much”; right anchor: “like very much”). They also had to explain the reasons that led them to appreciate or not the product.

### 2.5. Statistical Data Analysis

Analysis of variance (ANOVA), Tukey’s Honest Significant Difference (HSD) multiple comparison test (*p* < 0.05), Principal Component Analysis (PCA), word cloud and Partial Least Squares (PLS) regression were performed using XLSTAT software (ADDINSOFT, Paris, France).

For each sensory descriptor, ANOVA was performed with the product and panellist as the main effects, as well as their interaction, to investigate the performance of the panel and determine which descriptors can significantly discriminate the products [50]. The model used was as follows: descriptor intensity = panellist + product + panellist*product + error (random panellist effect). The ability of the panel to discriminate products was measured by the F product, and the panel was discriminating if the product effect was significant. Panel agreement was assessed by the panellist*product interaction: if the interaction was significant (*p* < 0.05), the panel disagreed. Finally, the Root of the Mean Square of Error (RMSE) indicated the repeatability of the panel: the smaller the RMSE was, the greater the panel was repeatable. Then, for each descriptor, a series of contrast tests were carried out to compare the mean intensity of each product with the mean of all the products (Grand mean (Gmean)). A summary table was used to present the results of these contrast tests: “+” or “−” signs were added next to means significantly above or below the Gmean (*p* < 0.05), respectively. Only significantly different means have been presented to improve the readability of the table.

Principal Component Analysis (PCA, covariance) was performed on the mean intensity for each sensory descriptor to highlight differences between the gels. The descriptors of texture and taste/aroma were represented on two separate PCAs.

Moreover, two additional ANOVAs and Tukey HSD tests were carried out, on the one hand, to assess the effect of heat treatment and the cultivar type on the intensity notes for the taste/aroma descriptors and the hedonic score, and on the other, to identify also the effects of formulation (absence or presence of salt and/or oil). The first model did not include the gels with added salt and/or oil and was as follows: intensity or hedonic score = panellist + cultivar + heat treatment + cultivar*heat treatment + error. The second model was focused on the gels made with cultivar 3 concentrate: intensity or hedonic score = panellist + salt + oil + heat treatment + salt*oil + salt*heat treatment + oil*heat treatment + error. These analyses were performed after calculating the average of the two repetitions for the sensory profiling data for each panellist and each product.

Partial Least Squares (PLS) regression analysis was carried out to determine the correlation between the sensory profile results (exclusion of non-discriminant and texture descriptors) and the hedonic score of the 12 gels.

In addition to the PLS, a word cloud representing the most frequently cited terms was created from the terms cited by consumers to explain what they liked or disliked in the different products (the word size was proportional to the frequency of citation). The first step consisted of synthesising whole sentences into keywords and grouping together words with the same semantic origin. Words describing the intensity level of descriptors (“too much”, “not enough”, or “very”) were not considered. The second step consisted of determining the occurrence frequency for each word, i.e., the percentage of consumers that cited this word to explain the hedonic score.

## 3. Results and Discussion

### 3.1. Sensory Profiling

#### 3.1.1. Performance of the Panel

The analyses showed that 14 of the 17 descriptors were used in a discriminating manner by the panel (Table 4). Three descriptors (yeast, broth and mushroom) did not make it possible to differentiate the products. They were well perceived in the gels but at an equivalent intensity from one product to another. They will be retained for subsequent analyses to obtain a complete evaluation of the sensory properties of the studied gels. For 10 of the 14 discriminating descriptors, the panellist*product interaction was significant, and the agreement between the subjects on these descriptors was not perfect. Nevertheless, for these descriptors, the F product was significant, which means that despite some disagreement, the observed differences are valid. Further training for these descriptors might reduce the disagreements and therefore increase the F product. The mean RMSE of the discriminant descriptors was 1.33. The 5 descriptors for which the panellists were the least repeatable were sticky, thick, cereal, foamy and green note, although the F product remained significant, and therefore, the descriptors were interpretable. Therefore, the trained panel was sufficiently effective to allow for the flavour characterisation of gels made with faba bean protein concentrates.

#### 3.1.2. Effects of the Heat Treatment and Formulation on the Gel Texture

**Figure 1:**
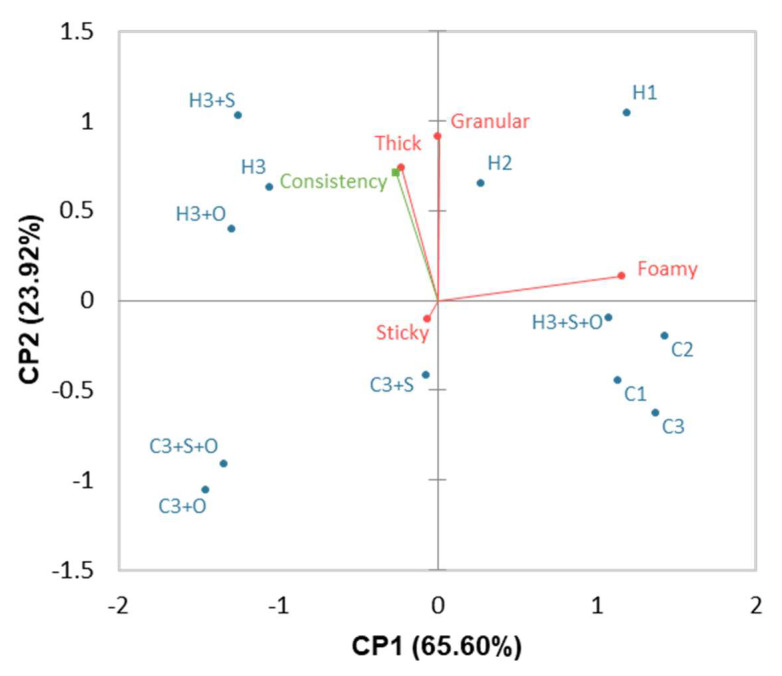
Biplot representation of the PCA (covariance) of the texture sensory descriptors (variables in red) and the consistency measured by back-extrusion (supplementary variable in green) of the 12 gels defined in Table 1.

The PCA presented in Figure 1 summarised 89.52% of the texture differences between the gels. The foamy descriptors strongly contributed to component 1, which alone explained 65.60% of the differences between the products. The thick and granular descriptors were carried out for component 2 of the PCA, which explained 23.92% of the differences between products. The short length of the sticky vector was in accordance with its lowest F product among the discriminating descriptors (Table 4). The orthogonality of the vectors representing, on the one hand, the foamy descriptor and, on the other hand, the thick and granular descriptors indicated that these descriptors were independent. Moreover, the thick and granular descriptors were very close on the PCA.

The gels made with crude protein concentrates (C1, C2 and C3) exhibited higher foaming sensory properties than those that were heated (H2 and H3). Heat processing should decrease the solubility of proteins due to their denaturation and reduce the foaming capacities [51,52]. Surprisingly, H1 had a foamy intensity similar to that of C1. Moreover, the addition of salt decreased the foaming intensities of the gels (C3+S, C3+S+O and H3+S), except for H3+O+S, which was foamier than H3. At low salt concentrations, foaming properties should increase, whereas, at high concentrations, they should decrease [53]. The gels with oil were less foamy except for H3+O+S, although the addition of oil should reduce the foaming properties [54]. Not all results were consistent with those of previous studies, especially for H1 (no change in foaming properties with heat treatment) and H3+O+S (unexpected foaming properties with the addition of salt and oil). These differences could be explained by the interaction between xanthan gum and proteins that could modify the foaming intensity of the gels. Indeed, the presence of xanthan gum in soy protein isolate increased the foaming property and stability compared to those of soy protein isolate alone [55].

The gels were relatively well discriminated for component 2: the gels made with heated concentrates (H) were located at the top section of the plot, whereas the gel containing crude concentrates (C) and H3+S+O appeared at the bottom. The gels with heated concentrates were characterised by high intensities of thickness and granulometry. When the temperature increases, protein denaturation occurs, and more protein aggregates are formed. The aggregates have a higher effective volume fraction than the individual molecules, which increases the consistency of the gels [56]. The gels with only the addition of NaCl were thicker due to the salting-in effect. At low salt concentrations, negatively charged chloride ions may interact with positively charged proteins, thereby decreasing electrostatic repulsion between proteins and increasing the formation of aggregates [57]. However, the addition of oil decreased the thickness. Oil may interact with hydrophobic protein sites, which may limit protein–protein interactions and the formation of aggregates [58]. Finally, the consistency (Table 1) and thickness were almost overlaid on the plot, which confirmed that the back-extrusion method was well appropriated to characterise the thick sensory descriptor of the gels. Moreover, the granular descriptor was very characteristic of the gels made with heated concentrates. It was not possible to obtain heated concentrates with a particle size as close as that of the crude concentrates. The use of industrial materials such as a more adapted mixer and/or a more selective sieve should allow for a reduction in the particle size.

#### 3.1.3. Effects of the Cultivar and Heat Treatment on the Gel Flavour

**Figure 2:**
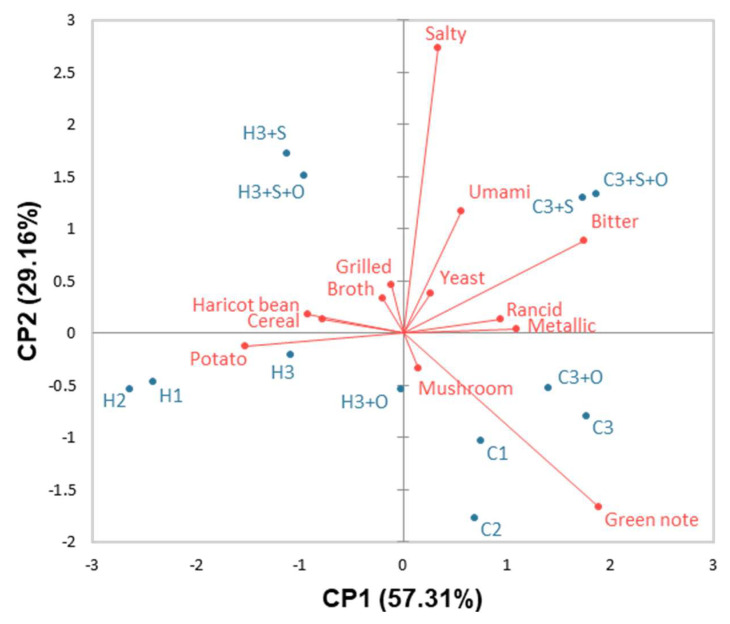
Biplot representation of the PCA (covariance) of the taste and aroma sensory descriptors of the 12 gels defined in Table 1.

To comprehensively characterise the flavour differences among the 12 gels, PCA was performed on the mean of the 3 taste and 10 aroma descriptors (Figure 2). The first component (57.31%) separated the gels characterised by green note, rancid, metallic and bitter descriptors in the right section of the plot from the gels described with haricot bean, cereal and potato aromas. The salty and umami intensities were more related to component 2 (29.16%). Grilled, broth, yeast and mushroom descriptors were too close to the PCA centre to be considered. Some descriptors have already been used to describe faba bean samples, such as fresh pea, grassy, yeast-like, grilled and bitter [31,33,34,35]. In our study, the green note descriptor gathered different aromas such as peas, French beans, asparagus, green legumes and grass (Table 2). All the aromas and tastes have already been used to describe pulse flavours [13,49].

The C gels were located in the right section of the plot. All of these gels exhibited high green note intensities, rancid and metallic notes, and bitterness. The ANOVA showed that bitterness and metallic aroma were significantly (*p* < 0.0001) cultivar dependent (Table 5). The gels made with concentrate from cultivar 3 exhibited higher bitterness, and the gels with the concentrate from cultivar 2 were less perceived as metallic (Table 6). The green, rancid and metallic notes are due to the presence of small volatile compounds originating mostly from free fatty acid enzymatic oxidation (lipoxygenase (LOX)) or autoxidation (high temperature) and free amino acid degradation [13,14]. However, the metallic perception originated from both volatile and non-volatile compounds (Table S2, Supplementary Materials). The metallic flavour could also be due to the oxidation of salivary proteins by minerals and oxidation-related aldehydes related to odourant lipids [28]. The bitterness in pulses is related to non-volatile compounds, including saponins, phenolic compounds, alkaloids, peptides and free amino acids [13,59]. Finally, the different sensory properties highlighted among cultivars could reveal differences in cultivar type, field and storage conditions or process transformation [14].

Concerning the H gels, they were located In the left section of the PCA and described by potato, cereal and haricot bean aromas. The heat treatment of concentrate as the main effect was highly significant for 8 descriptors, including green note, bitter, potato, metallic, cereal, rancid, haricot bean and broth (Table 5). This treatment decreased the green note, bitter, metallic and rancid perceptions and enhanced the potato, cereal, haricot bean and broth intensities (Table 6). Moreover, the cultivar*heat treatment interaction was significant (*p*-value = 0.046) only for the green note and showed that, although heat treatment significantly decreased the green note for all the gels, it was even stronger for the gels made with the concentrate from cultivar 2 (Figure S2, see Supplementary Materials). During the heating of faba bean concentrates in wet conditions, Maillard reactions could occur in the presence of reducing sugars and free amino acids and promote the formation of new compounds associated with cooked, roasted or cereal-like flavours [60,61]. The heated concentrates exhibited lower quantities of free amino acids and sugars (saccharose, glucose, fructose and galactose) than the crude concentrates (data not shown), suggesting that Maillard reactions could appear and lead to the development of roasted aromas. However, many studies have shown that the concentration of hexanal (grassy note) or (*E*,*E*)-octa-3,5-dien-2-one (vegetable, hay and earth aromas) increased in faba beans during heat treatment due to the autoxidation of free fatty acids without considering the concentrations of molecules responsible for the grilled notes [62,63,64]. This suggested that haricot bean, cereal, potato and broth aromas should be concentrated enough to partially mask green, rancid and metallic notes. Moreover, the haricot bean aroma was attributed to the heat treatment and also depended on the cultivar type (Table 5). The concentrate from cultivar 2 was more characterised by this aroma (Table 6). The decrease in bitterness during the heat process could be explained by many factors. Free amino acids may be converted into Maillard products and decrease bitter perception. Moreover, non-volatile compounds potentially responsible for pulse bitterness, such as saponins, alkaloids or phenolic compounds (flavonoids and proanthocyanidins), could be degraded at high temperatures and reduce the bitterness intensity of heated concentrates [65,66,67]. Moreover, the compounds responsible for the green, metallic and rancid notes and bitterness could be degraded during the heat treatment or masked by the formation of new ones. The control of storage conditions after the heating process is critical to avoid the formation of unwanted volatile compounds in faba bean concentrates [31,63].

#### 3.1.4. Effect of the Formulation on the Gel Flavour

As expected, gels with added salt were perceived as saltier. More surprisingly, the gels with the addition of salt were also perceived as more umami than those without salt, whereas all the gels contained the same quantity of added MSG (Umami: F salt 19.07, *p*-value < 0.0001; ANOVA (panellist + salt + oil + treatment + salt*oil + salt*treatment + oil*treatment + error); data not shown). MSG has been shown to have an enhancer effect on saltiness in soup [68]. The green (F salt = 5.42, *p*-value = 0.021) and grilled (F salt = 8.40, *p*-value = 0.004) notes were significantly affected by salt addition. The addition of salt decreased the green note intensity but increased the grilled note intensity. First, the presence of salt could modify the protein–volatile compound interactions; only the non-binding volatile compounds (the released ones) might contribute to the product flavour [69,70,71,72]. A hypothesis could be that the 3-isopropyl- and 3-isobutyl-2-methoxypyrazines previously identified in faba bean concentrates and partially responsible for the green note [32] are less hydrophilic than hexanal (grassy aroma) and could interact with proteins in the presence of salt, which decrease their release and contribute less to the green aroma intensity. Second, taste could influence aroma perception [73]. This suggested that green notes could be reduced by salt and/or that salt could enhance grilled aroma, which decreased the green note perception. Finally, the addition of salt into legume-based products allowed us to modify the perception of umami and some aromas. However, the use of oil in the formulation had no effect on the descriptor intensities.

### 3.2. Consumer Liking

#### 3.2.1. Effects of the Cultivar, Heat Treatment and Formulation on Gel Appreciation

**Figure 3:**
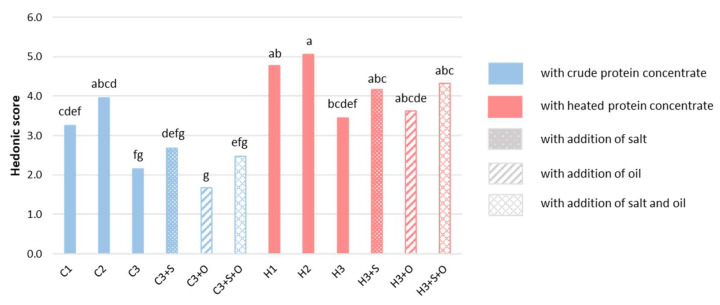
Hedonic score means of the 12 gels (80 consumers). Different letters indicate significant differences considering the same analysis (α = 0.05, Tukey HSD test). The gels are defined in Table 1.

The hedonic score means are presented in Figure 3. The gels made with crude or heated cultivar 2 concentrate (C2 and H2) were preferred over the other concentrates (C1, C3, H1 and H3). Moreover, the appreciation means for crude concentrates were lower than those for heated concentrates (2.70 ± 0.81 compared to 4.23 ± 0.63). According to the ANOVA, the gel appreciation depended significantly on both the cultivar and heat treatment (Table 5). Cultivars 1 and 2 and the heat treatment increased the hedonic score (Table 6).

The formulation effects on the liking of products were studied with the second ANOVA model (data not shown). The addition of oil to the gel did not change the product preferences (F oil = 0.27, *p*-value = 0.604). However, the addition of salt had a positive effect on product appreciation (F salt = 19.75, *p*-value < 0.0001). This positive effect of salt is well known and has already been demonstrated in beef patties containing textured soy [74].

#### 3.2.2. Taste and Aroma Properties Guiding Gel Acceptability

**Figure 4:**
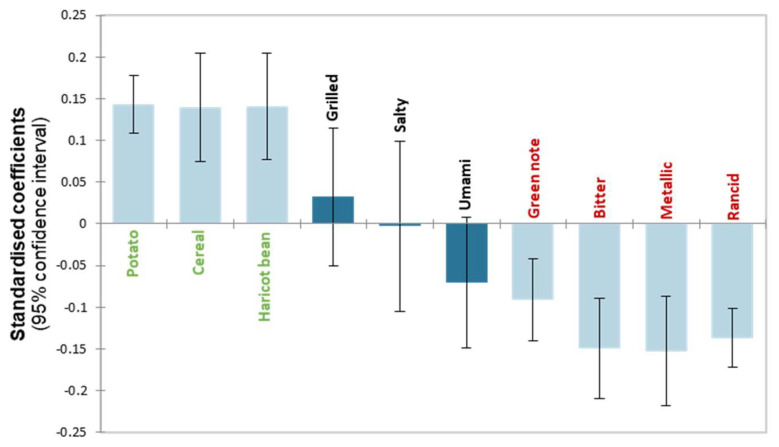
95% Jack-knife confidence intervals of the descriptor coefficients in the PLS regression (one significant PLS component). The coefficients in black were not significant; those in green contributed positively to the hedonic score, whereas those in red contributed negatively.

To identify the sensory descriptors guiding the acceptability of the products, the data from the sensory profile and the consumer study were combined. The objective was to determine the descriptors that most decreased the acceptability of the products. A PLS regression was performed to explain the hedonic appreciation scores (dependent variable) using the intensities of these descriptors (explanatory variables) [75]. The texture descriptors and the yeast, broth and mushroom non-discriminant aroma descriptors were excluded from the prediction. The PLS regression model (R^2^ = 0.855; Q^2^ (cum) = 0.819 for a single component) indicated significant descriptors (Figure 4). The descriptors grilled, salty and umami were not significant. Potato, cereal and haricot bean descriptors contributed positively to the appreciation of the gels. Conversely, green note, bitter, metallic and rancid descriptors contributed negatively to the appreciation of gels. These penalising descriptors were already identified as off-flavours in pulses [13]. According to regression Model (1), the cereal descriptor contributed more to the appreciation of the product than haricot bean or potato. The product with heated concentrates, especially H1 and H2, exhibited higher intensities of these aromas (Gmean, Table 4). However, the presence of metallic and rancid descriptors in the gels decreased consumer acceptability more than the green note and bitter characteristics. The C3 gel and its derivatives showed high metallic and rancid intensities that could be attributed to the cultivar (Table 4 and Table 6). The bitterness was more characteristic of the gel made with the protein concentrate from cultivar 3 and the crude concentrate gels (Table 6). Although the green note was previously identified as an off-note, in this study, it contributed less to product rejection. For example, C2 exhibited a higher green note (+2.14 from Gmean) than C3 (+0.63 from the Gmean) and was more appreciated by consumers (hedonic score means of 4.0 and 2.1, respectively). Indeed, this gel was characterised by very low bitter intensity.

The PLS regression model showed that the appreciation of the gels depended on a combination of several sensory factors. Even if the sensory characteristics decreasing acceptability have been clearly identified, it is important to note that a higher intensity than the average for one of these negative descriptors should not necessarily be prohibitive. For example, unpleasant descriptors could be slightly present in the gel, whereas pleasant descriptors could be more intense. This combination should not reduce product acceptability due to a probable phenomenon of compensation. In the context of the formulation of novel legume-based products, it is important to consider all of these sensory perceptions, not only one. For example, during varietal selection, it would be regrettable to eliminate a cultivar due to an intense green note higher than the average; this cultivar may not be characterised by important bitterness, metallic and rancid notes and/or could exhibit potato, cereal and haricot bean aromas (naturally after heating), which could limit the effect of the green note on product liking.

Finally, to improve faba bean acceptability, different strategies could be proposed. The selection of a cultivar that does not contain the molecules responsible for the bitterness, green, metallic and rancid off-flavours should be imperative while keeping in mind that it is necessary to consider combinations of molecules rather than the effects of single molecules. Moreover, this study shows that the wet heat treatment improved consumer acceptability by promoting the formation of pleasant notes such as potato, cereal and haricot bean aromas and reducing the perceptions of unpleasant flavours. Although the heat treatment was beneficial in terms of the flavour quality, it may influence other properties, in particular, the texture and the functional properties of faba bean protein concentrates. These consequences should be considered when formulating new products. Finally, the results are related to a salty gel food model and could be different for other food applications.

(1) Hedonic score=3.29+0.22 Potato+0.39 Cereal+0.31 Haricot bean−0.09 Green note−0.18 Bitter−0.31 Metallic−0.32 Rancid 

**Figure 5:**
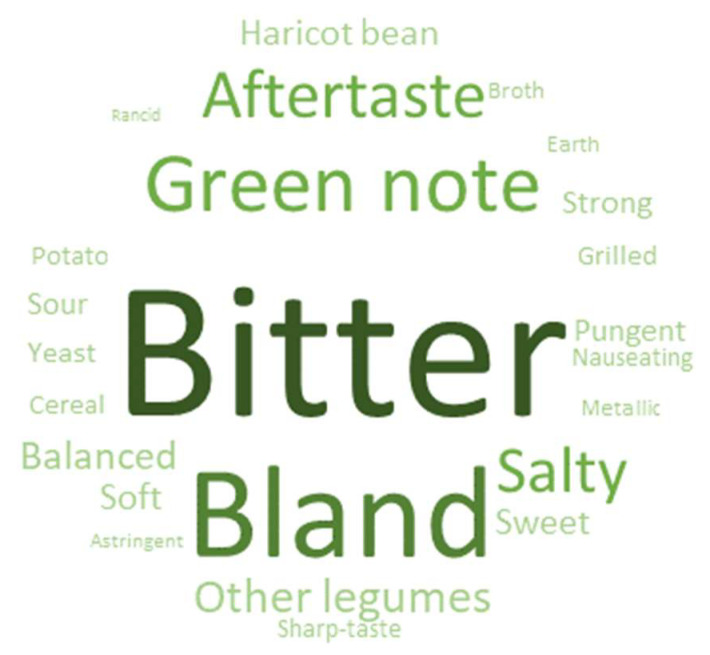
Word cloud based on the 24 perception classes that explained the hedonic score of the 12 gels.

To complete the results from the PLS regression, the reasons given by consumers to explain the appreciation evaluation were represented in the form of a word cloud composed of words whose size depends on the frequency of citation (Figure 5). The words most frequently cited appear with a larger size. A total of 96 different keywords were cited and grouped among 24-word classes, including balanced, bland, bitter, strong, green note, salty, aftertaste, sweet, soft, sour, haricot bean, potato, cereal, grilled, yeast, broth, pungent, other legumes, sharp-taste, metallic, earth, astringent, rancid and nauseating. The sensory descriptors resulting from the sensory profile partly inspired the formation of these classes, particularly when choosing the names of the classes grouping the terms mentioned by consumers. Bitter was the most cited word with 208 citations which showed that this characteristic was well perceived and considered in the consumer assessment. Bitterness is a flavour that is identified well by consumers, even without training, which explains why they had no difficulty in identifying it as a factor. The word bland was more related to aroma perception. Indeed, the consumers often said that the gels were salty or bitter but also bland (for example: “Bland, no particular flavour, leaves some bitterness in the mouth”). The green note, aftertaste and salty taste were cited 78, 60 and 56 times, respectively (data not shown). The green note consisted mostly of pea, green legume and vegetal notes. Consumers did not all agree with each other; some liked the green note, while others did not. The aftertaste was described as “persistent”, “unpleasant”, or “pleasant”, but no reference to a specific taste or aroma was made. A recommendation for the next sensory profile of pulses would be to evaluate the descriptor before and after swallowing to identify those responsible for the aftertaste. Finally, the other negative perceptions already identified by the PLS regression, including metallic (5 citations) and rancid (2 citations), were rarely mentioned. During the sensory profiling, the descriptors metallic and rancid required much training, whereas the bitterness perception, for example, was already acquired. This suggests that even if consumers detected metallic and rancid notes, they were not familiar enough with these words to mention them. Moreover, it was probably easier to identify these perceptions under pungent (18 citations), sour (17 citations), sharp-taste and nauseating (both 10 citations) descriptors.

## 4. Conclusions

This study made it possible to establish the sensory profile of protein concentrates, crude or heated, from three cultivars of fava beans in a gel model. The gels made with crude concentrates exhibited green, yeast and mushroom aromas. However, some of them were characterised by rancid and metallic notes and bitterness related to the cultivar diversity. The heating of these concentrates modified their flavour with the promotion of potato, cereal, haricot bean and broth notes and a reduction in the green, metallic and rancid notes and bitterness. However, the functional properties of the concentrates were modified and should be considered according to the formulation needs. The addition of salt modified the perception of green and grilled notes, whereas the oil had no effect. The correlation between the sensory profile and the consumer test results highlighted the descriptors reducing the acceptability of the gels and, therefore, potentially the acceptability of food products made from the studied protein extracts. The rancid, metallic and bitter perceptions contributed more to gel rejection than the green notes. However, it is more important to focus on all the perceptions and their impacts on the flavour than on one descriptor alone. This study was carried out on a gel which represented a simple food model system allowing for validation of concepts but was not very realistic. The next step would be to test the acceptability of these legume protein extracts in applications closer to commonly consumed foods, for which the purpose was to incorporate these fractions to improve their nutritional qualities, such as biscuits, bread, plant-based meat substitutes, prepared meals, etc. Finally, the cultivar type and the heat treatment should be simple strategies to increase the flavour of faba bean concentrate and improve the consumer acceptability of this legume. Other strategies to reduce off-flavours are under development, such as fermentation, grain sprouting, development of cultivars lacking LOX to limit the unpleasant aroma of pulses. Combinations of various strategies should lead to faba bean extracts being highly appreciated by consumers.

## Figures and Tables

**Table 1:** Name, composition and consistency (N.mm) of the 12 studied gels.

Gel Samples	Type of Concentrate	Protein Concentrate (g)	Xanthan Gum (g)	MSG ^1^ (g)	Water (g)	NaCl (%)	Oil (%)	Consistency ^2^ (N.mm)
C1	P1	20	1.0	0.6	79	-	-	1.80 ^h^ ± 0.08
H1	HP1					-	-	2.62 ^de^ ± 0.04
C2	P2					-	-	1.89 ^gh^ ± 0.21
H2	HP2					-	-	3.17 ^bc^ ± 0.17
C3	P3					-	-	2.12 ^fgh^ ± 0.01
C3+S						0.25	-	2.86 ^cd^ ± 0.01
C3+O						-	3.0	2.13 ^fg^ ± 0.06
C3+S+O						0.25	3.0	2.40 ^ef^ ± 0.06
H3	HP3					-	-	3.30 ^b^ ± 0.02
H3+S						0.25	-	3.70 ^a^ ± 0.19
H3+O						-	3.0	3.27 ^b^ ± 0.14
H3+S+O						0.25	3.0	3.44 ^ab^ ± 0.06

^1^ MSG: Monosodium glutamate. ^2^ Values are expressed as mean ± standard deviation (*n* = 3); different letters indicate significant differences considering the same analysis (α = 0.05%, Tukey HSD test).

**Table 3:** Sociodemographic characteristics and pulse consumption frequency of consumers.

		Panel	French Population ^1,2^
Sex	Women (%)	49	52
	Men (%)	51	48
Age (year)	20–39 (%)	33	31
	40–59 (%)	34	35
	60 and over (%)	33	34
Level of education	<Baccalaureate * (%)	18	29
	≥Baccalaureate (%)	82	40
Pulse consumption frequency	1 to 3 times a month (%)	10	-
	≥once a week (%)	90	48

* Baccalaureate: French high school diploma. ^1^ 2018 figures, INSEE (National Institute of Statistics and Economic Studies). ^2^ 2021 press release about the CREDOC (Research Centre for the Study of the Conditions of Life) survey, Terres Univia (Vegetable Oils and Proteins Inter-branch Organisation, France).

**Table 4:** Results of ANOVA (product + panellist + panellist*product) to examine panellist performance (significant *p*-values are in bold (α = 0.05)) and comparison of the gel mean with the Grand mean (Gmean).

Descriptor	Product		Panellist*Product		RMSE *	Gmean **	Gel Mean											
	F	*p*-Value	F	*p*-Value			C1	H1	C2	H2	C3	C3+S	C3+O	C3+S+O	H3	H3+S	H3+O	H3+S+O
Foamy	14.65	**<0.001**	1.44	**<0.01**	1.68	2.90	+1.09	+1.15	+1.44		+1.27		−1.62	−1.44	−1.00	−1.02	+1.03	−1.20
Green note	12.74	**<0.001**	0.90	0.79	2.06	3.39	+0.89	−1.23	+2.14	−1.03	+0.85	+0.63			−0.71	−1.13		−1.19
Granular	11.64	**<0.001**	1.39	**<0.01**	0.96	0.84	−0.49	+1.29		+0.38	−0.40	−0.43	−0.59	−0.59	+0.52	+0.61		
Bitter	10.75	**<0.001**	1.91	**<0.001**	1.27	2.66		−1.33	−0.52	−1.67	+0.94	+0.90	+1.05	+0.97				
Salty	10.29	**<0.001**	2.25	**<0.001**	1.27	2.68	−0.71	−0.61		−0.61	−0.83	+1.30	−0.60	+1.29	−0.59	+1.35	−0.64	+1.13
Potato	6.73	**<0.001**	2.19	**<0.001**	1.17	1.88		+1.14		+1.07	−0.81	−0.54	−0.53	−1.00				
Metallic	6.31	**<0.001**	1.34	**<0.05**	1.15	1.43		−0.66		−0.90	+0.62	+0.52	+0.70					
Thick	6.12	**<0.001**	0.91	0.77	1.58	3.95				+0.48	−0.72		−0.69		+0.50	+1.17		+0.62
Cereal	4.79	**<0.001**	0.45	1.00	1.66	1.62		+0.34		+0.37		−0.45	−0.48	−0.47	+0.39	+0.37		+0.48
Umami	4.21	**<0.001**	1.74	**<0.001**	1.14	1.72		−0.66	−0.55			+0.89		+0.79				
Rancid	4.04	**<0.001**	2.17	**<0.001**	0.98	0.90		−0.53		−0.52	+0.63			+0.78	−0.47			
Haricot bean	3.98	**<0.001**	1.71	**<0.001**	1.18	2.31				+0.90	−0.62					+0.71		
Grilled	2.06	**<0.05**	0.65	1.00	1.02	1.08			−0.33							+0.25		+0.32
Sticky	1.96	**<0.05**	1.30	**<0.05**	1.47	3.68						+0.54				+0.59	−0.57	
Yeast	1.20	0.29	1.87	**<0.001**	1.15	1.65			−0.49			+0.61						
Broth	0.95	0.49	1.00	0.50	1.44	1.73										+0.49		
Mushroom	0.81	0.62	1.49	**<0.001**	1.15	1.29												

* Root-Mean-Square Deviation. ** For each gel, the mean was compared with the Grand mean (Gmean) and was included in the table only if it was either significantly lower (“−“ in red) or higher (“+” in blue) (*p* < 0.05).

**Table 5:** Results of ANOVA (panellist + cultivar + heat treatment + cultivar*heat treatment) to examine the taste/aroma descriptor intensities and liking score of 6 gels (C1, C2, C3, H1, H2 and H3) (Table 1). Significant *p*-values are in bold (α = 0.05).

Descriptors	Panellist		Cultivar		Heat Treatment		Cultivar*Heat Treatment	
	F	*p*-Value	F	*p*-Value	F	*p*-Value	F	*p*-Value
Green note	10.61	**<0.0001**	2.57	0.081	74.29	**<0.0001**	3.17	**0.046**
Bitter	16.69	**<0.0001**	18.88	**<0.0001**	31.55	**<0.0001**	0.03	0.966
Salty	24.80	**<0.0001**	0.34	0.709	0.15	0.697	0.43	0.653
Potato	15.12	**<0.0001**	2.90	0.059	30.91	**<0.0001**	0.13	0.880
Metallic	17.04	**<0.0001**	4.93	**0.009**	26.77	**<0.0001**	0.15	0.858
Cereal	49.08	**<0.0001**	0.19	0.830	16.95	**<0.0001**	0.29	0.751
Umami	19.97	**<0.0001**	2.73	0.070	1.46	0.230	1.72	0.185
Rancid	6.74	**<0.0001**	1.14	0.324	13.08	**<0.001**	0.82	0.443
Haricot bean	32;35	**<0.0001**	7.72	**0.001**	15.27	**<0.001**	0.00	1.000
Grilled	59.25	**<0.0001**	2.05	0.135	2.61	0.110	0.60	0.553
Yeast	22.22	**<0.0001**	0.81	0.449	0.02	0.896	1.43	0.265
Broth	50.07	**<0.0001**	0.18	0.837	4.15	**0.044**	0.56	0.572
Mushroom	11.71	**<0.0001**	0.48	0.621	0.71	0.402	0.58	0.562
Hedonic	5.18	**<0.0001**	26.36	**<0.0001**	43.04	**<0.0001**	0.38	0.685

**Table 6:** Results of the Tukey HSD comparisons of 6 gels (C1, C2, C3, H1, H2 and H3; Table 1) for the descriptors with significant cultivar or heat treatment effects and/or cultivar*heat treatment interaction (*p*-value < 0.05; Table 5). Significant differences between horizontal groups for each effect and interaction are indicated by differences in letters (α = 0.05). Shade cells correspond to the absence of significant effect and/or interaction (*p*-value > 0.05; Table 5).

Descriptors	Cultivar			Heat Treatment		Cultivar*Heat Treatment					
	1	2	3	No	Yes	1*No	2*No	3*No	1*Yes	2*Yes	3*Yes
Green note				4.69 ^a^	2.40 ^b^	4.28 ^a^	5.53 ^a^	4.24 ^a^	2.16 ^b^	2.36 ^b^	2.68 ^b^
Bitter	1.94 ^b^	1.56 ^b^	3.05 ^a^	2.76 ^a^	1.61 ^b^						
Potato				1.46 ^b^	2.77 ^a^						
Metallic	1.23 ^ab^	0.90 ^b^	1.56 ^a^	1.67 ^a^	0.79 ^b^						
Cereal				1.43 ^b^	1.99 ^a^						
Rancid				1.13 ^a^	0.39 ^b^						
Haricot bean	2.22 ^b^	2.86 ^a^	2.05 ^b^	2.02 ^b^	2.73 ^a^						
Broth				1.56 ^b^	1.85 ^a^						
Hedonic	4.01 ^a^	4.51 ^a^	2.79 ^b^	3.12 ^b^	4.42 ^a^						

## Data Availability

The data presented in this study are available on request.

## Supplementary Materials

The following supporting information can be downloaded at: https://www.mdpi.com/article/10.3390/foods11193018/s1. Figure S1: Similarity rates obtained for gels made with different concentrations of monosodium glutamate (MSG) (from 0 to 0.6%) by the sensory panel (21 panellists); Figure S2: Average intensity scores for green note according to the cultivar type and heat treatment (results from the ANOVA model: green note intensity = panellist + cultivar + heat treatment + cultivar*heat treatment + error); Table S1: Results of the Tukey HSD comparisons of the taste intensities (salty, bitter and umami) evaluated by the 21 trained panellists on 4 gels (C2, H2, C3 and H3—no addition of MSG) with and without a nose clip; Table S2: Percentage of panellists that detected a metallic perception with and without a nose clip.
