# Supplementary material for: Heat Treatment, Cultivar and Formulation Modify the Sensory Properties and Consumer Acceptability of Gels Containing Faba Bean (Vicia faba L. minor) Protein Concentrates
Source: Foods. 2022 Sep 28;11(19):3018. doi: 10.3390/foods11193018 (PMC9562209; doi:10.3390/foods11193018)
Supplement: Supplementary file 1 [file foods-11-03018-s001.zip › foods-1858350-supplementary.pdf]

Heat Treatment, Cultivar and Formulation Modify the Sensory Properties and Consumer Acceptability of Faba Bean (*Vicia faba* L. *minor*) Protein Concentrates

Adeline Karolkowski [1,2], Christophe Martin [1,3], Emilie Bouzidi [2], Jean-François Albouy [2], Loïc Levavasseur [2], Loïc Briand [1], Christian Salles [1,*]

[1] CSGA (Centre des Sciences du Goût et de l'Alimentation), CNRS, INRAE, Institut Agro, Université de Bourgogne-Franche Comté, F-21000 Dijon, France
[2] Groupe Soufflet (Ets J. Soufflet), 10400 Nogent-sur-Seine, France
* Correspondence: christian.salles@inrae.fr (C.S); Tel.: +33-806-930-79

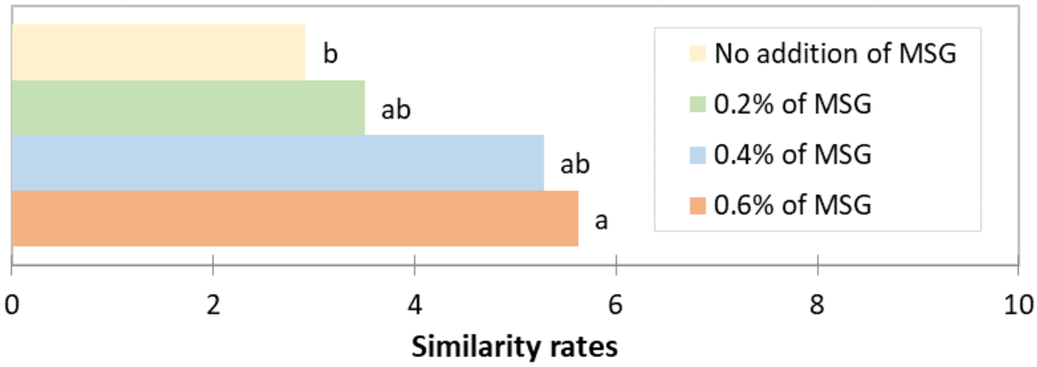

Figure S1. Similarity rates obtained for gels made with different concentrations of monosodium glutamate (MSG) (from 0 to 0.6%) by the sensory panel (21 panellists). The gels were formulated with 20 g of industrial crude concentrate, 1 g of xanthan gum and 79 g of water. The scale ranged from 0 (far from the reference (protein concentrate)) to 10 (close to the reference). Different letters indicate significant differences considering the same analysis (α = 0.05%, Tukey HSD test).

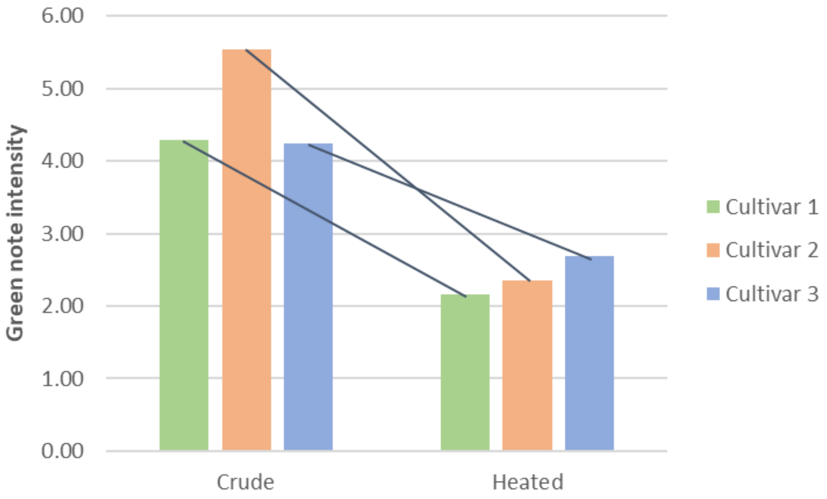

Figure S2. Average intensity scores for green note according to the cultivar type and heat treatment (results from the ANOVA model: green note intensity = panellist + cultivar + heat treatment + cultivar*heat treatment + error). Following the heat treatment, the intensity decreased more strongly for cultivar 2 than for the other cultivars. This difference in the effect of heat treatment depending on the cultivar explained the observed interaction (see black lines in the figure).

Table S1. Results of the Tukey HSD comparisons of the taste intensities (salty, bitter and umami) evaluated by the 21 trained panellists on 4 gels (C2, H2, C3 and H3 – no addition of MSG) with and without a nose clip. Significant differences between groups are indicated by different letters.

	C2		H2		C3		H3		p-value
Nose clip	*with*	*without*	*with*	*without*	*with*	*without*	*with*	*without*	
Salty	a	a	a	a	a	a	a	a	0.914
Bitter	b	b	b	b	a	a	ab	ab	< 0.001
Umami	a	a	a	a	a	a	a	a	0.918

For each gel, the product aroma (perceived without a nose clip) did not affect the taste intensities.

Table S2. Percentage of panellists that detected a metallic perception with and without a nose clip.

	**With a nose clip** (perception of non-volatile compounds)	**Without a nose clip** (perception of both volatile and non-volatile compounds)
Percentage of trained panellists that detected a metallic perception	69%	100%
